# CyREST: Turbocharging Cytoscape Access for External Tools via a RESTful API

**DOI:** 10.12688/f1000research.6767.1

**Published:** 2015-08-05

**Authors:** Keiichiro Ono, Tanja Muetze, Georgi Kolishovski, Paul Shannon, Barry Demchak

**Affiliations:** 1 Department of Medicine, University of California, San Diego, CA, 92093-0688, USA; 2 Imperial College London, London, SW7 2AZ, UK; 3 Bioconductor, Boston, MA, 02125, USA; 4 Fred Hutchinson Cancer Research Institute, Seattle, WA, 98109, USA

**Keywords:** Workflow, Reproducibility, Cytoscape, Service Oriented Architecture, Interoperability, REST, Resource Oriented Development, Microservice

## Abstract

As bioinformatic workflows become increasingly complex and involve multiple specialized tools, so does the difficulty of reliably reproducing those workflows. Cytoscape is a critical workflow component for executing network visualization, analysis, and publishing tasks, but it can be operated only manually via a point-and-click user interface. Consequently, Cytoscape-oriented tasks are laborious and often error prone, especially with multistep protocols involving many networks.

In this paper, we present the new cyREST Cytoscape app and accompanying harmonization libraries. Together, they improve workflow reproducibility and researcher productivity by enabling popular languages (e.g., Python and R, JavaScript, and C#) and tools (e.g., IPython/Jupyter Notebook and RStudio) to directly define and query networks, and perform network analysis, layouts and renderings. We describe cyREST’s API and overall construction, and present Python- and R-based examples that illustrate how Cytoscape can be integrated into large scale data analysis pipelines.

cyREST is available in the Cytoscape app store (http://apps.cytoscape.org) where it has been downloaded over 1900 times since its release in late 2014.

## Introduction

Because of its robust network analysis and visualization capabilities coupled with its vibrant user and developer community, Cytoscape 3 has become a tool of choice for studying large network-oriented *omics data sets on common workstations and for publishing results. However, even as Cytoscape ^1^ is well positioned to handle customized *omics workflows, bioinformaticians’ need to quickly and efficiently create complex, varied, and repeatable workflows exceeds the capabilities of Cytoscape’s existing automation features. At the same time, bioinformaticians have embraced a class of highly flexible tools consisting of fully fledged programming environments (e.g., IPython/Jupyter Notebook ^2^, RStudio, and MATLAB) coupled with programming languages (e.g., Python and R) and highly capable and flexible bioinformatic libraries.

Inasmuch as these tools address the data collection and analysis portions of typical bioinformatic workflows, Cytoscape complements them by addressing visualization, additional analysis, and network publication. To date, combining these tools with Cytoscape has seen only limited success, largely because of the limitations of Cytoscape’s automation interfaces and its point-and-click user interface. Consequently, this integration has been labor intensive, inconvenient, and often unrepeatable, particularly as the complexity of analysis and visualization processing increases.

**Figure 1.**
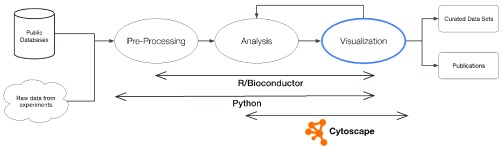
Cytoscape working with other components to create bioinformatic workflows.

We created the cyREST Cytoscape app to enable automated access to the Cytoscape network and visualization models directly from within these tools, thereby exposing Cytoscape visualization, analysis, and publishing features in complex, varied, and reproducible bioinformatic workflows as shown in Figure 1.

**Figure 2.**
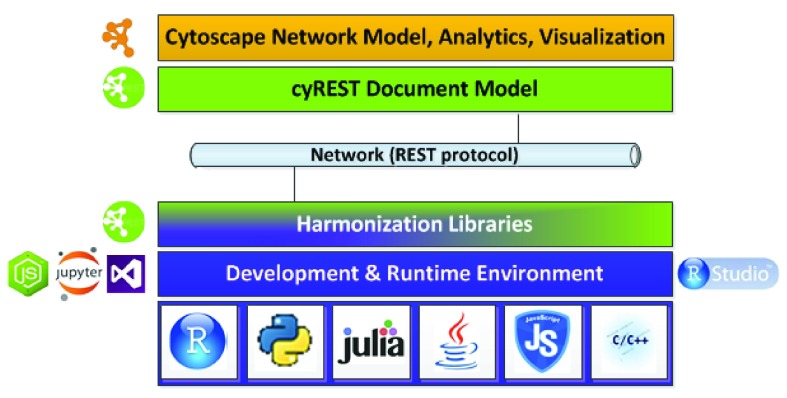
Relationship of Cytoscape to Bioinformatics-oriented Tools and Languages, where cyREST is in green. Harmonization libraries are green and blue, representing an interface between workflow code and cyREST.

cyREST transforms Cytoscape into a REST-based microservice ^3^ easily callable by workflows coded in REST-enabled languages such as Python, R, and Java. It is complemented by language-specific libraries that simplify Cytoscape access and harmonize native data models with Cytoscape’s network model as shown in Figure 2. (REST ^4^ is short for Representational State Transfer.)

cyREST complements Cytoscape’s existing Command Line Tool, where cyREST operates on Cytoscape’s data and Command executes Cytoscape commands. Since its original beta release date in late 2014, cyREST has been downloaded over 1900 times.

In this paper, we explain how cyREST relates to existing Cytoscape-oriented automation solutions, and then describe the design and use of cyREST itself. Finally, we give examples of cyREST’s use from applications written in Python (using IPython Notebook) and R (using RStudio).

### Existing tools

Several Cytoscape apps and plugins tackle tool interoperability and workflow reproducibility challenges ( Table 1), most notably Cytoscape 3’s Command core module, but also including plugins deprecated along with Cytoscape 2.

**Table 1.** Existing Cytoscape apps to control Cytoscape from scripts.

Application Name	Description	Supported Version
**Command** ^5^	A domain specific language to automate simple tasks in Cytoscape	3.x and 2.x
**Cyrface** ^6^	An interface to call R packages from Cytoscape	3.x
**ScriptingEngine** ^8^	Exposes Cytoscape API to scripting engines for JVM (JavaScript, Jython, JRuby, etc.)	2.x
**CytoscapeRPC** ^15^	Plugin to call low-level Cytoscape API through XML-RPC	2.x
**RCytoscape** ^17^	Bioconductor package to call Cytoscape from R using CytoscapeRPC	2.x

Cytoscape 3’s Command Line Tool ^5^ facilitates task automation via its own domain specific language, which provides access to high-level Cytoscape functions using a separate REST server within Cytoscape. While Command can execute individual commands (e.g., for loading and applying layouts) and sequences of commands (as scripts), it has no provision for accessing the network, style, and visualization information available through cyREST. Command is a complement to cyREST, where the combination greatly improves interoperability between Cytoscape and workflow-capable external tools, which contribute looping and control flow. A workflow can intermix Command and cyREST calls without conflict – they address different capabilities within Cytoscape.

A notable alternative to cyREST is the Cyrface app ^6^, which allows R and Bioconductor ^7^ functions to be executed *from* Cytoscape 3, with the results returned *to* Cytoscape 3 – the opposite of a cyREST call. While this approach enables Cytoscape to act as the workflow orchestrator, it requires that a target application act as a server, which often requires idiosyncratic and complex support for each target application. So far, this approach has been taken only for interfacing to R.

Numerous approaches to interoperability were implemented as ScriptingEngine ^8^-based plugins for Cytoscape 2, now deprecated. Such plugins were created for executing scripts written in languages (e.g., JRuby ^9^, Jython ^10^, Groovy ^11^, Clojure ^12^, and JavaScript ^13^). While these scripts had full access to Cytoscape’s comprehensive public API, their tight coupling to the Cytoscape runtime made them difficult to write, debug, and maintain. Because they were built on top of the Java virtual machine (JVM) and shared Cytoscape’s JVM, they had little access to increasingly capable and standardized third party native libraries (e.g., SciPy ^14^ for Python). By contrast, the cyREST approach allows control of Cytoscape by best-of-breed tools and languages running independently as separate processes and leveraging best-of-breed native libraries. Conversely, while plugin implementations could interact with the user via dialog boxes directly within Cytoscape 2, scripts executing in separate processes run within their own windows, disconnected from Cytoscape 3.

Similar to the cyREST approach, the CytoscapeRPC plugin ^15^ enabled independent scripts (e.g., Python) to create, query, and modify networks and visual styles in Cytoscape 2, but using an XML-RPC ^16^ protocol instead of REST. Given the rapid adoption of REST conventions and supporting infrastructure, use of XML-RPC is becoming less common. Notably, RCytoscape ^17^ is a Bioconductor package that leveraged CytoscapeRPC to enabled R applications to control Cytoscape 2. For Cytoscape 3, RCytoscape has been replaced by the RCy3 package in Bioconductor release 3.2, which leverages cyREST instead and is described below.

## What is cyREST?

cyREST is a Cytoscape app that exposes Cytoscape network-related data structures and publishing functionality as a microservice callable via a REST protocol by external tools and languages. To date, it offers over 113 API calls, as documented at http://idekerlab.github.io/cyREST, where each API call accepts JSON-encoded values ^18^ and returns JSON-encoded results.

Given that most modern tools and languages can call JSON-oriented REST services either directly or through well-vetted libraries, cyREST enables near-universal access to Cytoscape. However, such tools and languages often define data structures well tuned for use with their own specialized libraries that manipulate network-oriented data. To ease and accelerate the programming process, cyREST provides harmonization libraries designed to make calling cyREST natural and native within a tool or language. Harmonization libraries are described below.

Note that while cyREST enables Cytoscape to act as a service, it is intended to serve only one client application at a time, where the client and Cytoscape run on the same workstation. Cytoscape itself remains capable of working on a single Cytoscape session at a time and maintains a visible window accessible to a user – Cytoscape does not operate in so-called headless mode. As a result, a client application is free to implement a workflow that intentionally sets up a network within Cytoscape so that a user can work further with it.

This section describes both the cyREST design and implementation and the implementation of harmonization libraries. It then presents example workflows created by combining standard data analysis tools with Cytoscape/cyREST.

### Design

cyREST is a Cytoscape app that exposes the Cytoscape network data model to external tools and languages. It presents an API based on principles of REST, as do other popular biology-related data services, including those provided by EBI ^19^. As a result, cyREST leverages REST facilities in existing tools and languages already built and vetted for use with other REST-based services. The definition and packaging of individual API functions takes advantage of lessons learned in building similar interfaces for Cytoscape 2.

cyREST APIs represent all Cytoscape data objects and functions as resources according to principles of Resource-oriented Design (ROD) ^20^. Data objects include networks, tables, and Visual Styles. Functions include applying layout algorithms on networks, updating Visual Styles, and performing statistical analysis. Under REST and ROD, each resource is encoded as a URL where hierarchy is represented as segments within the URL. For example, the URL http://localhost:1234/v1/tables/count can be decomposed into a REST server ( http://localhost), port number (1234), an API version (v1), a resource (tables), and an attribute of the resource (count). So, this URL represents the count of global tables maintained by Cytoscape. Table 2 shows a sampling of resources available under the http://localhost:1234/v1 URL, with a more comprehensive list in the cyREST document at http://idekerlab.github.io/cyREST).

**Table 2.** Examples of Cytoscape data resources exposed by cyREST.

URL Segment	Data Resource
**networks**	list of all networks (as networkId)
**networks/networkId/edges**	edges within network networkId
**networks/networkId/nodes**	nodes within network networkId
**networks/networkId/tables**	tables within network networkId
**networks/networkId/views**	views of network networkId
**networks/networkId/groups**	node groupings within network networkId
**session**	session-wide attributes (e.g., name)
**styles**	visual styles that can apply to networks
**styles/styleName/mappings**	values of visual properties for style styleName

cyREST follows ROD recommendations for sensible mappings between CRUD operations (create, read, update, and delete) and HTTP operations (POST, GET, PUT, DELETE) on data objects. Unless otherwise specified in the cyREST documentation, all HTTP operations accept or return values encoded as JSON. For example, GET http://localhost:1234/v1/networks returns a list of networkIds in an array (e.g., [1,2,3]). GET http://localhost:1234/v1/networks/networkId returns all nodes, edges, tables, and other data relating to network networkId in the Cytoscape.js ^21^ JSON format.

For functions, ROD provides less guidance for CRUD/HTTP mappings or URL encoding. cyREST addresses this by grouping actions under http://localhost:1234/v1/apply (using GET operations) as illustrated in Table 3.

**Table 3.** Examples of Cytoscape function resources exposed by cyREST.

URL Segment	Function Resource
**edgebundling/networkId**	apply edge bundling to edges in network networkId
**fit/networkId**	fit network networkId to its window
**layouts/algorithm/networkId**	apply a layout algorithm to network networkId
**styles/styleName/networkId**	apply visual style styleName to network networkId

### Implementation

cyREST is implemented as a Cytoscape app written in the Java programming language. It uses the Jersey JAX-RS ^22^ library to implement the RESTful API, and provides access to data object and function operations as calls to public Cytoscape APIs. Under REST, each client request is phrased as an HTTP command (e.g., GET http://localhost:1234/v1/networks HTTP/1.1) and the reply is returned as a JSON structure.

cyREST uses an embedded Grizzly HTTP server to receive and process client requests, where each HTTP request’s URL is mapped to a method in a resource manager class created by cyREST and registered with Grizzly. Each resource method declares the URL it services. When Grizzly receives a REST request, it calls the resource function registered for the URL, which calculates and returns a REST reply. For example, the NetworkResource defines a function that returns the number of Cytoscape networks in the current session, shown in the code fragment below. Note that the fragment defines its associated HTTP command, URL, and JSON output via Java annotations.



                        @Path(
                        "/v1/networks"
                        )

                        public class 
                        NetworkResource 
                        extends

                            AbstractResource {
  @GET
  @Path(
                        "/count"
                        )
  @Produces(MediaType.APPLICATION_JSON)

                          public 
                        String getNetworkCount() {

                            // Call Cytoscape to get count of network
        - return value as COUNT

                            return

                                getNumberObjectString(JsonTags.COUNT,
        networkManager.getNetworkSet().size());
}
                    


As with most Cytoscape apps, cyREST is initialized in its cyActivator function, which creates resource classes that reference all Cytoscape APIs to be used in servicing client requests. These include factories and managers for networks, network views, visual mapping, layout algorithms, groups, tables, sessions, and others.

The default HTTP port for cyREST is 1234, which can be changed by creating or modify the Cytoscape rest.port property (via Cytoscape’s Edit | Preferences | Properties dialog). Note that security-conscious workstations should firewall the cyREST port to prevent unintended outside access.

To test for the availability of a cyREST server, use an Internet browser to view the URL http://localhost:1234/v1/, which returns JSON-formatted version information similar to:



                        {

                        	"apiVersion"
                        :
                        "v1"
                        , 
                        
	"numberOfCores"
                        :4, 
                        
	"memoryStatus"
                        : {

                        	       "usedMemory"
                        :517, 
                        
	       "freeMemory"
                        :1445, 
                        
	       "totalMemory"
                        :1963, 
                        	
	       "maxMemory"
                        :6917
	}
}
                    


Each cyREST function is exercised and validated before release by a suite of JUnit-based tests.

### Harmonization libraries

While most programming languages make calling REST APIs and composing or parsing JSON simple, the data returned by cyREST may not be organized efficiently for ease of use in a particular language or with that language’s libraries. To maximize programmer productivity, we provide harmonization libraries (see Figure 2) to perform efficient cyREST calls on one hand, and present an interface easily used by programmers on the other hand. To date, we provide harmonization libraries for Python and R, and we expect to produce others.

### py2cytoscape harmonization library for Python

The Python programming language has become popular among scientists and data analysts because of its rich collection of open source data analysis packages and a large developer community. It is an excellent tool for data cleansing, manipulation, analysis, and visualization; its igraph ^23^, NetworkX ^24^, and graph-tool ^25^ packages are useful components in network data analysis workflows. In a workflow, it functions well as a glue that connects multiple heterogeneous computing resources, public databases, and private data files to build data analysis pipelines on workstations and computing clusters.

We created the py2cytoscape library to enable Python-based workflows to easily incorporate Cytoscape functionality by wrapping Python calls to cyREST and performing automatic translations between these packages’ data structures and cyREST’s JSON. For example, the following code creates a new Cytoscape network by using py2cytoscape calls, and replaces 16 lines that would be necessary when calling cyREST directly – see https://github.com/idekerlab/py2cytoscape/blob/develop/README.md for the direct cyREST calls.



                        from py2cytoscape.data.cyrest_client 
                        import

                            CyRestClient

cy = CyRestClient()
network = cy.network.create(name=
                        ’My Network’
                        , 
    collection=
                        ’My  network  collection’
                        )
print(network.get_id())
                    


py2cytoscape is open source and is available from the PyPI repository ( https://pypi.python.org/pypi/py2cytoscape).

Note that py2cytoscape provides a widget that renders a network in cytoscape.js JSON format and then visualizes the network interactively within a Jupyter/IPython Notebook ^26^ document, an example of which is at http://nbviewer.ipython.org/github/idekerlab/py2cytoscape/blob/develop/examples/New_wrapper_api_sample.ipynb.

### RCy3 harmonization library for R

R is a particularly important platform for biologists because of the complimentary Bioconductor library. We are collaborating with the Bioconductor group to produce the RCy3 harmonization library for R ^27^, which leverages cyREST to realize native R network visualization, analysis, and publishing functions. Its igraph, graph ^28^, and RBGL ^29^ packages are useful components for network data analysis workflows.

### Sample workflows

A typical workflow performs data acquisition and integration, analysis, network visualization, and publishing. Often, these steps are performed one at a time by humans executing one discreet tool after another, possibly resulting in high labor costs, low throughput, high error rates, and an inability to reproduce the workflow reliably. In contrast, Figure 1 shows a workflow orchestrated by external tools such as Python and R, which interact with Cytoscape to perform parts of the workflow. As supplementary material, we provide downloadable sample workflows that incorporate and demonstrate cyREST functionality using py2cytoscape and RCy3 harmonization libraries.

Note that Cytoscape/cyREST is designed to run on the same workstation as the workflow that calls it – Cytoscape maintains its own application window, and workflows may find advantage in soliciting users directly within Cytoscape.

### Python examples

Our Python-based sample workflows are simple reflections of real world data analysis and visualization pipelines (see Figure 1) and use standard Python packages as much as possible. They are located in https://github.com/idekerlab/cy-rest-python and are viewable using the nbviewer web application ( http://nbviewer.ipython.org/) in Jupyter Notebook format.

Some Python packages are more capable or faster than equivalent Cytoscape functions, so the examples use them instead of calling Cytoscape. For example, Pandas ^30^ prepares and analyzes data by using NumPy ^31^ and SciPy library for processor-intensive tasks such as community detection.

The examples use the py2cytoscape harmonization libraries to demonstrate efficient cooperation between Python workflows by using NetworkX, igraph, and Cytoscape to integrate and visualize data generated in external tools. They show:

- Data import from multiple data sources (remote/local)
- Reformat and integration
- Statistical network analysis
- Visualization

For instance, the “Import KEGG pathways from web service” example demonstrates a typical biological data integration and visualization process involving KEGG databases ^32^:

- Send a disease name query to the KEGG API
- Filter the result and reformat it
- Import disease pathway data directly from the KEGG database
- Visualize pathway data in Cytoscape
- Embed the result as an interactive network diagram in the Jupyter Notebook

This workflow is simple to do with Cytoscape – the alternative would be a custom program or manual, file based operations that are hard to reproduce. With this workflow script, collaborators or reviewers can easily execute the same process on their environment, which is essential for reproducible scientific research.

### R examples

For network analysis and visualization, igraph is an important and much used package by R users, and our sample R workflows ( https://github.com/idekerlab/cy-rest-R) use it to complement the graph analysis features in Cytoscape.

**Figure 3.**
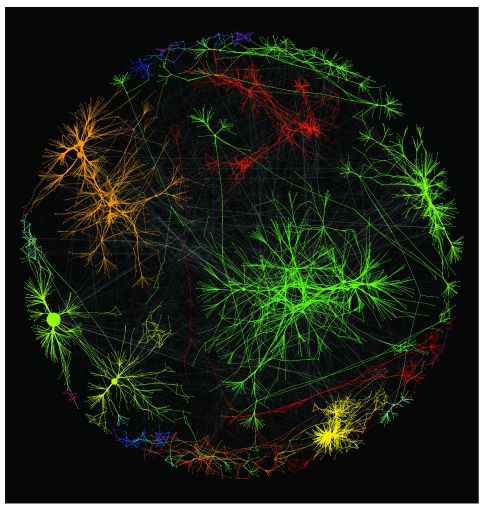
Yeast network ^37^ visualization with sample R script and Cytoscape.

In our Workflow 1 example, we scripted typical network visualization techniques using igraph’s graph analysis functions and Cytoscape’s data visualization features. First, we used igraph to detect community structure using a fast greedy modularity optimization algorithm ^33^, and we calculated basic statistics of the network, including PageRank ^34^ and betweenness centrality ^35^. Our R code calls Cytoscape to create the resulting network, set properties for both layout and visual mapping, and generate an interactive network visualization. Output of this workflow helps users to visually understand the basic structure of the network (see Figure 3, which shows community structures color coded and used as weights for the Kamada-Kawai layout algorithm ^36^).

In many cases, users apply an automatic layout algorithm early in a workflow to visually check the overall structure of a network. However, such layouts are often based on a simple force simulation and tend to produce uninformative “hairballs.” Our example illuminated network sub structures by using a community detection algorithm and igraph’s statistical analysis algorithms and visual styling. The alternative would be manual operation of both R and Cytoscape, which is laborious and error prone even for proficient users, and which is not reusable for subsequent networks.

## Future development

In this paper, we demonstrated Python- and R-based workflow examples. In the near future, we expect to demonstrate cyREST usage in MATLAB and JavaScript (via Node.js ^38^). While the cyREST and Command apps implement different automation features, we expect to unify the two APIs through a common implementation library in the Cytoscape core in the next Cytoscape release. Existing Cytoscape implementations manage a single Cytoscape session on behalf of a single user, can produce screen visualizations, and can potentially solicit user input even while under the control of cyREST. Future versions of Cytoscape will run headlessly and service multiple sessions simultaneously.

## Summary

Cytoscape is a highly popular desktop application for network biology analysis, visualization, and publication. The cyREST app extends Cytoscape into the realm of reproducible and high volume bioinformatic workflows by exposing a RESTful API that recasts Cytoscape as a visualization and rendering microservice. Using cyREST, data acquisition and analysis workflows previously limited to low quality (if any) visualizations can now leverage Cytoscape’s substantial library of network layouts, visualization features, and rendering options. Similarly, cyREST’s seamless integration with research and publication tools such as IPython/Jupyter Notebook improves individual researcher productivity by avoiding the need to manually operate Cytoscape.

Because it presents a RESTful interface, cyREST benefits can be realized in workflows built in most modern programming languages, and represents a significant contribution to productivity and reproducibility in data driven biology.

## Software availability

CyREST software is available from the Cytoscape App Store: http://apps.cytoscape.org/apps/cyrest

Latest source code of cyREST: https://github.com/idekerlab/cyREST

Full REST API v1 document: http://idekerlab.github.io/cyREST/

Py2cytoscape is in beta and is installable from PyPI repository: https://pypi.python.org/pypi/py2cytoscape

Py2cytoscape source code: https://github.com/idekerlab/py2cytoscape

Python sample workflows in Jupyter Notebook format: https://github.com/idekerlab/cy-rest-python

R sample workflows: https://github.com/idekerlab/cy-rest-R

License for cyREST, py2cytoscape, and all example workflows: MIT: http://opensource.org/licenses/MIT

RCy3 source code: https://github.com/tmuetze/Bioconductor_RCy3_the_new_RCytoscape
