# Supplementary material for: CyREST: Turbocharging Cytoscape Access for External Tools via a RESTful API
Source: F1000Res. 2015 Aug 5;4:478. [Version 1] doi: 10.12688/f1000research.6767.1 (PMC4670004; doi:10.12688/f1000research.6767.1)
Supplement: Peer review history

# Peer review: Referee response for version 1

**DOI:** 10.5256/f1000research.7270.r9846

**Published:** 2015-08-20

**Authors:** Guanming Wu

**Affiliations:** 1 Ontario Institute for Cancer Research, Toronto, ON, Canada

The authors describe a Cytoscape app, CyREST, which exposes core Cytoscape functions as REST APIs for external software components to process network related data sets in automatic and reproducible workflows built using almost any programming languages. Users of workflows can visualize network data in Cytoscape via its powerful visualization features. The accompanied harmonization libraries for Python and R make the use of CyREST much easier and simpler. The manuscript is well organized, and the described app should be highly valuable for users working with big data related to networks for analysis and visualization.

Minor comments:

 

1. Figure 1: The difference between R/Bioconductor and Python is not clear to me. The double-arrowed line for Python is longer than R/Bioconductor, but I am not sure what extra is covered by Python. Python is a full-fledged programming language and capable for anything. Should some specific packages listed for Python? Further, probably the line for Cytoscape should be extended to cover two boxes for Curated Data Sets and Publications.
2. Figure 2: Will it be better to call “Network (REST protocol)” just as “REST Protocol” to avoid confusing with other uses of "network" in the manuscript?
3. What is the development status of RCy3? It will be nicer to indicate its status somewhere. From the manuscript, it seems that this is still in work-in-progress?

Typos:

 

1. In abstract: e.g. Python ***and R***, JavaScript, and C#
2. There are several typos in this page: https://github.com/idekerlab/cy-rest-R, e.g. **Cytoscaep**, **Lesons**, etc.

I have read this submission. I believe that I have an appropriate level of expertise to confirm that it is of an acceptable scientific standard.

# Peer review: Referee response for version 1

**DOI:** 10.5256/f1000research.7270.r9848

**Published:** 2015-08-10

**Authors:** Sergey Nepomnyachiy

**Affiliations:** 1 Department of Computer Science & Engineering, Polytechnic Institute of New York University, Brooklyn, NY, USA

The authors present a Cytoscape app that turns it into a REST server. Most core functionality of Cytoscape can now be triggered by external agents using HTTP requests, rather than by point-and-clicking UI. This enables workflow automation and distribution that was hardly possible before. The fact that the protocol is HTTP based, allows working with it programmatically using any modern language. CyREST comes with libraries for Python and R that make the communication with Cytoscape through REST API even simpler, by enveloping the calls in methods and wrapping the returning JSON in objects.

Perhaps in the future work section the authors could shed some light on the plans to allow users extending the set of REST handlers. The virtues of Cytsocape are beyond the core functionality and currently there is no way for a Cyto-app developer to map her own functions to REST (those are hard-wired in the code of CyREST app). A simple registry for binding 3rd party Cyto-app functions to URLs at runtime could bring the community to bring their on plug-in REST. 

Possible typos

page 6:

The default HTTP port for cyREST is 1234, which can be changed by creating or ***modify*** the Cytoscape rest.port property

page 7:

This workflow is simple to do with Cytoscape – the alternative would be a custom program ***or manual, file based operations*** that are hard to reproduce.

I have read this submission. I believe that I have an appropriate level of expertise to confirm that it is of an acceptable scientific standard.
